# Temporary Housing for Older People: Addressing Housing Insecurity to Promote Aging in the Right Place

**DOI:** 10.1093/geroni/igab046.1131

**Published:** 2021-12-17

**Authors:** Shreemouna Gurung, Shelby Elkes, Atiya Mahmood, Holly Lemme, Gelareh Modara, Emily Lam, Maria Juanita Mora, Sarah Canham, Rachel Weldrick

**Affiliations:** 1 Simon Fraser University, Vancouver, British Columbia, Canada; 2 University of Utah, Salt Lake City, Utah, United States

## Abstract

The Aging in the Right Place (AIRP) project is a multi-year, multi-city partnership grant on aging, housing insecurity and homelessness. This paper presents findings from provider/staff interviews (N=5) at a Temporary Housing Program (THP) serving older people experiencing (or at risk of) homelessness (OPEH) in Vancouver, Canada. The researchers sought to understand the strengths and weaknesses of the program, scale-up (i.e., policies) and/or scale out impacts (i.e., on people and communities), as well as how the program promotes housing security and stability for OPEH. Narrative data reveals the program provided housing stability to OPEH by offering increased access to resources (food, pharmaceutical, transportation, social support and engagement). Additionally, through the promotion of client autonomy, privacy and security in their housing unit, the organization and staff work to support and foster AIRP among their clients and help to transform a temporary housing space into a secure home-type setting.

